# Low Serum 25-hydroxyvitamin D Level Does Not Adversely Affect Bone Turnover in Prepubertal Children

**DOI:** 10.3390/nu13103324

**Published:** 2021-09-23

**Authors:** Wojciech J. Bilinski, Lukasz Szternel, Joanna Siodmiak, Przemyslaw T. Paradowski, Krzysztof Domagalski, Grazyna Sypniewska

**Affiliations:** 1 Department of Orthopaedics, KoMed, Poddebice Health Center, 85067 Poddebice, Poland; 2 Department of Laboratory Medicine, Collegium Medicum, Bydgoszcz, Nicolaus Copernicus University, 87110 Torun, Poland; lukaszszternel@wp.pl (L.S.); asiapollak@wp.pl (J.S.); odes@cm.umk.pl (G.S.); 3 Department of Surgical and Perioperative Sciences, Division of Orthopedics, Sunderby Research Unit, Umeå University, Sunderby Central Hospital of Norrbotten, 90187 Luleå, Sweden; przemyslaw.t.paradowski@gmail.com; 4 Faculty of Health Sciences, Collegium Medicum, Bydgoszcz, Nicolaus Copernicus University, 87110 Torun, Poland; 5 Department of Immunology, Faculty of Biological and Veterinary Sciences, Nicolaus Copernicus University, 87110 Torun, Poland; krydom@umk.pl

**Keywords:** bone turnover markers, insulin-like growth factor 1, 25-hydoxyvitamin D, bone mineral accrual

## Abstract

Both vitamin D and insulin-like growth factor 1 (IGF-1) play essential roles in bone metabolism and may interact during prepubertal bone accrual. We investigated the association of low serum 25-hydroxyvitamin D (25(OH)D) (<20 ng/mL) with the circulating bone turnover markers, when compared to their interaction with IGF-1. Subjects and Methods: Serum 25(OH)D, IGF-I, P1NP (N-terminal propeptide of type I procollagen), and CTX-1 (C-terminal telopeptide of type I collagen) were measured, and the bone turnover index (BTI) was calculated in 128 healthy children, aged 9–11 years. Results: Mean 25(OH)D concentration was 21.9 ± 4.9 ng/mL, but in 30.5% of participants it was <20 ng/mL (<50 nmol/L). We observed a trend for higher P1NP (*p* < 0.05) and IGF-1 (*p* = 0.08), towards lower 25(OH)D in tertiles. Levels of P1NP in the lowest 25(OH)D tertile (<20 ng/mL) were the highest, while CTX and BTI remained unchanged. Additionally, 25(OH)D negatively correlated with IGF-1, while the correlation with P1NP was not significant. A strong positive correlation of IGF-1 with P1NP and BTI but weak with CTX was observed. Low 25(OH)D (<20 ng/mL) explained 15% of the IGF-1 variance and 6% of the P1NP variance. Conclusions: Low levels of 25(OH)D do not unfavorably alter bone turnover. It seems that serum 25(OH)D level may not be an adequate predictor of bone turnover in children.

## 1. Introduction

Optimal bone growth during childhood and adolescence is critical to reach a maximal strength and size of the skeleton. It is assumed that up to 90% of adult bone mineral content is achieved in early adulthood [1]. The higher the peak bone mass and bone mineral density, the slower the age-related bone loss, and the lower the risk of development of osteoporosis and subsequent fractures. The association of vitamin D status with bone health throughout childhood is subject to change, depending on age and pubertal stage. Studies have suggested a possible interaction between 25-hydroxyvitamin D and IGF-1 during prepubertal bone mineral accrual [2,3]. The key “bone trophic hormones”, growth hormones (GH) and IGF-1, are essential for achieving peak bone mass [4,5]. GH acts directly on bone cells and stimulates IGF-1 to increase bone formation through the enhancement of osteoblasts proliferation, differentiation, and activity [4]. While GH decreases the differentiation and function of osteoclasts, IGF-1 enhances the bone resorption by stimulating the osteoclasts activity [4]. Nevertheless, bone formation outweighs resorption, leading to an increase in bone mass. 

A sufficient level of circulating 25-hydroxyvitamin D enables the adequate synthesis of the active metabolite 1,25(OH)2D and ensures adequate plasma calcium concentration. Vitamin D is the key regulator of bone metabolism, and its deficiency contributes to a higher level of parathyroid hormone (PTH), leading to the enhancement of bone turnover. The active metabolite, 1,25(OH)2D, has indirect and direct effects on bone metabolism, which involves the control of the extracellular matrix proteins, synthesis by osteoblasts, mineralization, and stimulating RANK (receptor activator of nuclear factor kappa beta)-dependent bone resorption [6,7]. Measurement of 1,25(OH)2D3 has several methodological limitations; thus, vitamin D status is assessed by the determination of serum 25-hydroxyvitamin D concentration (25(OH)D), which reflects an individual’s vitamin D status from both endogenous and exogenous sources. However, this approach is not optimal, due to factors affecting the concentration of 25(OH)D, such as the rate of its metabolism and the levels of vitamin D-binding protein, which may differ between individuals. In accordance with the recent European Calcified Tissue Society Working Group Position statement, serum 25(OH)D concentrations should be above 12 ng/mL (30 nmol/L) at all ages to avoid rickets or osteomalacia, while vitamin D deficiency in the general population was defined as a 25(OH)D concentration below 20 ng/mL (50 nmol/L) [8].

With the currently established cut-off values, the occurrence of 25-hydroxyvitamin D concentrations below 20 ng/mL in children is high and exceeds 30% [8,9,10]. However, there is no common agreement on optimal circulating 25(OH)D concentration in growing children and its association with skeletal growth is not well established. Most of the guidelines define vitamin D insufficiency in children as serum 25(OH)D between 12–20 ng/mL (30–50 nmol/L) [11,12]. The level of 25(OH)D which may negatively affect bone mineralization and cause rickets is generally accepted as below 12 ng/mL (<30 nmol/L) and defined as a deficiency [11,12]. A previous observational study revealed that 25-hydroxyvitamin D concentration in childhood is not good enough predictor of peak bone mass in early adulthood [13]. Recent studies in adults also suggested that 25(OH)D alone may be a poor biomarker of bone health, not associated with bone mineral density or risk of fractures [14].

In an earlier prospective study, the influence of circulating 25(OH)D and IGF-1 on bone mineral content accrual (BMC) at different skeletal sites was examined in healthy prepubertal girls with a sufficient vitamin D status [2]. The results of this study indicated that vitamin D status and IGF-1 were significantly associated with bone mineral accrual during growth [2]. 

Assessment of serum bone formation and bone resorption markers, as surrogate indices reflecting the metabolism of the skeleton, is often applied in clinical practice. It was demonstrated that in prepubertal healthy children, bone turnover markers (bone alkaline phosphatase and CrossLaps) are positively related with IGF-1 [15]. However, in the other study, neither IGF-1 nor 25(OH)D correlated with the circulating markers of bone turnover P1NP (N-terminal propeptide of type I procollagen) and CTX-1 (C-terminal telopeptide of type I collagen) examined in prepubertal and pubertal children with sufficient vitamin D status [16]. Even though the high occurrence of vitamin D concentrations below 20 ng/mL (50 nmo/L) in European pediatric populations evidence seems insufficient to recommend a screening for vitamin D deficiency [17,18]. It is plausible that the association between IGF-I and markers of bone metabolism vary in children with low levels of 25(OH)D. We aimed to investigate the association of serum bone turnover markers (P1NP and CTX) with low 25(OH)D levels (<20 ng/mL), compared to their interaction with IGF-1.

## 2. Materials and Methods

### 2.1. Characteristics of Study Participants

The study involved 128 presumably healthy school children (66 girls and 62 boys), aged 9–11 years. Inclusion criteria for study participants were as follows: body mass index (BMI) centile ≥5, normoglycemia (fasting glucose <100 mg/dL; <5.6 mmo/L), no fractures within at least 2 months preceding the blood draw, normal physical activity, and no supplement or medicine intake that might affect their vitamin D levels or bone metabolism. To avoid potential bias, due to seasonal differences in sun exposure, the recruitment and blood collection process took place within one month. The general health of each child was evaluated by school nurses and a general practitioner on the day of blood collection. Pubertal status was recorded based on self-assessment reports. Tanner stage 1 (TS1) was reported by 86% of girls and 74% of boys, whereas 12% of girls and 18% of boys reported TS2. Only 1 girl and 5 boys reported TS3.

### 2.2. Laboratory and Anthropometric Measurements

Fasting blood was drawn to perform all the assays. Total 25-hydroxyvitamin D, IGF-I, intact-P1NP, and CTX-1 were measured in frozen serum samples. P1NP and CTX-I are recommended as reference markers by the International Federation of Clinical Chemistry and Laboratory Medicine (IFCC) and the International Osteoporosis Foundation for investigations in adults [19]. Concentrations of total 25(OH)D, i-P1NP, CTX-1, and IGF-1 were analyzed on an IDS-iSYS automated platform (Immunodiagnostic Systems Holding PLC, Boldon, UK). The method used for total 25(OH)D measurement is characterized by a very low cross-reactivity with 3-epi-25(OH)D3 and 3-epi-25(OH)D2 (<1%), particularly important when assessing 25-hydroxyvitamin D in children. The concentrations of total 25(OH)D were presented in ng/mL. The assays used for bone formation marker i-P1NP and bone resorption marker CTX show the intra-assay CV < 5%. All samples for i-P1NP determination were pre-diluted, whereas measurement of CTX-1 did not require dilution.

Anthropometric measurements and blood draws were performed on the same day. Age- and sex-specific BMI percentile values were calculated as previously described [17,20]. BMI percentile classifications were performed in accordance with the guidelines of the International Obesity Task Force: underweight BMI <5; overweight ≥85 and <95; obesity ≥95 percentile [17].

The bone turnover index (BTI) was calculated as previously described in detail [21,22]. The BTI value provides additional information on the bone turnover [21]. Positive BTI values indicate the predominance of bone formation, while a negative BTI value favors bone resorption [21]. The concentration of total 25(OH)D <20 ng/mL (<50 nmol/L) was accepted as an insufficiency, according to the recommendations for pediatric populations [11,12]. 

The study was conducted in accordance with the Declaration of Helsinki and was approved by the Collegium Medicum Bioethics Committee at the Nicolaus Copernicus University (KB 338/2015 annex 487/2019). All participants and their parents were thoroughly informed about all aspects of the study, and written informed consent was obtained.

### 2.3. Statistical Analysis

Descriptive statistics were used to define the characteristics of the study group. Continuous variables were presented as a means ± standard deviation (Gaussian distribution) or medians with 25th (Q1) and 75th (Q3) percentiles (non-Gaussian distribution). Comparisons between the groups were done using the Student’s *t*-test for normally distributed variables and the Mann-Whitney U-test for non-normally distributed variables. The associations between variables were analyzed by the Pearson or Spearman correlation. Linear regression analysis was applied to assess variables of significance for the prediction of P1NP and CTX variance in the study group. Gender and BMI percentile were adjusted in the models. Statistical analysis was performed using SPSS (versions 20 and 27, Armonk, NY, USA) software. Statistically significant *p*-value was <0.05.

## 3. Results

Demographic characteristics and the concentrations of 25-hydroxyvitamin D, bone turnover markers, and IGF-1 are presented, dependent on gender (Table 1). Both groups were comparable, in terms of BMI centile, frequency of obesity (10.6 vs. 12.9%, respectively), the levels of 25(OH)D, and bone markers (P1NP, CTX). It is worth noting, boys had significantly lower IGF-1 levels and bone turnover index. Mean value of 25(OH)D in the study participants was much below <30 ng/mL (<75 nmol/L). In addition, the frequency of low 25(OH)D <20 ng/mL (<50 nmol/L) was slightly lower among boys than among girls (25.8 vs. 34.8%). 

Characteristics of study participants after stratification into tertiles of 25(OH)D is presented in Table 2. We observed a significant trend (*p* = 0.048) for higher levels of bone formation marker (P1NP) and a trend for IGF-1 (*p* = 0.08) towards lower 25(OH)D concentrations. CTX levels and bone turnover index remained unchanged.

When study participants were stratified into tertiles by their baseline IGF-1 concentrations, we observed a significant trend for higher levels of P1NP (*p* < 0.001), bone turnover index (*p* = 0.045), and height (*p* = 0.043), as well as a trend for higher body mass (*p* = 0.056) towards higher IGF-1 levels. CTX and 25(OH)D concentrations did not change significantly (Supplementary Material, Table S1). Bone turnover index reflected the predominance of bone formation over resorption, particularly at the highest IGF-1 levels.

Correlation analysis of the whole study group showed a strong positive association between IGF-1 and P1NP, and a weak but significant positive association with CTX, BTI, and body mass (Table 3). IGF-1 and P1NP were weakly associated with participants’ height (r = 0.228, *p* = 0.01 and r = 0.195, *p* = 0.027, respectively); no correlation was observed for CTX. Of note, we did not find correlations between P1NP, CTX and BMI percentile (r = −0.015, *p* = 0.865 and r= −0.155, *p* = 0.080, respectively). By contrast, 25(OH)D was negatively related with IGF-1 and a trend toward a negative relationship with P1NP was observed. We did not find association between 25(OH)D and bone resorption marker CTX. 

Further, we developed the linear regression models for the prediction of P1NP, CTX, and IGF-1 variation by low 25-hydroxyvitamin D concentration (<20 ng/mL). All models were adjusted for BMI percentile and gender. It was not possible to design a model for BTI variation. Only the models with P1NP and IGF-1 were statistically significant. Low 25(OH)D concentration explained 15% of the IGF-1 variance and 6% of the P1NP variance (Table 4). 

## 4. Discussion

Childhood and adolescence are particularly important periods for accruing bone because the skeleton undergoes a rapid change. In both girls and boys, bone mass increases substantially, as a result of bone modeling, leading to the modification of the size and shape of bones. During early skeletal development, bone resorption and bone formation processes are uncoupled [23]. An earlier prospective study in prepubertal girls with sufficient vitamin D status (35.3 ± 9.8 ng/mL) showed that 25(OH)D and IGF-1 exert a significant impact on bone mineral content (BMC) accrual, assessed by DXA [2]. Another fact worth mentioning is that the association of 25(OH)D with bone accrual was shown to be negative and weaker than positive association of IGF-1 with BMC [2]. In another prospective study, bone mineral content accrual over 12 months was assessed in children and adolescents, in relation to PTH and 1,25(OH)2D levels [24]. Secondary analyses in this study investigated the association of bone formation (bone-alkaline phosphatase) and resorption markers (urinary deoxypyridinoline) levels with 25(OH)D in a subset of the study group. It was demonstrated that greater bone accrual in children and adolescents was associated with higher PTH, higher 1,25(OH)2D, and higher bone formation marker levels, but not with 25(OH)D concentration. 

We investigated the association of serum 25-hydroxyvitamin D and IGF-1 with the bone turnover markers during prepubertal growth in healthy children. Average concentration of serum 25(OH)D in the study participants was slightly over 20 ng/mL (21.9 ± 4.9 ng/mL). However, 30.5% of all subjects had insufficient vitamin D status (<20 ng/mL; <50 nmol/L). Only two participants had 25(OH)D below 12 ng/mL (<30 nmol/L), generally defined as a deficiency. We found that low levels of 25(OH)D (<20 ng/mL; <50 nmol/L) did not adversely alter bone turnover in prepubertal children, and this is an important finding of our study. Low levels of 25(OH)D were associated with significantly higher bone formation marker (P1NP) concentrations, with concomitantly unchanged bone resorption marker levels. Consequently, the bone turnover index (BTI) remained unchanged and indicated that, in children with low vitamin D levels, bone formation still exceeded bone resorption.

The correlation between 25(OH)D and P1NP only neared statistical significance and no correlation of 25(OH)D with CTX was observed. Similarly, DeBoer et al. did not find correlations between 25(OH)D and bone markers in children and adolescents [24]. However, they noted a highly significant positive correlation between 1,25(OH)2D and bone markers (*p* < 0.001).

In children with insufficient vitamin D status, the influence of low 25(OH)D on IGF-1 variance was modest, and low 25(OH)D also had a limited effect on bone formation marker variance (6%). By contrast, we found a strong positive correlation of IGF-1 with bone formation marker (*p* < 0.001) and indicated that increasing IGF-1 levels had a significant positive effect on bone turnover index. The positive impact of IGF-1 on bone mass, at several skeletal sites, was observed by others in early pubertal children with hypovitaminosis D, while no correlation was noted between 25(OH)D levels and bone mineral density [24]. Also, a weak but significant correlation of 1,25-dihydroxyvitamin D level with bone mineral content was found [24].

Next, we showed a weak, significant negative correlation between 25-hydroxyvitamin D and IGF-I. In the previous study by Breen et al., performed in prepubertal girls with sufficient vitamin D status, a negative but nonsignificant relationship between 25(OH)D and IGF-1 was noted [2]. As suggested, it is possible that higher levels of IGF-I concentrations lead to lower concentrations of 25(OH)D because IGF-I stimulates the hydroxylation of 25(OH)D to the biologically active 1,25(OH)2D metabolite [2,4]. It was reported that vitamin D status may affect the liver’s secretion of IGF-1 and the expression of IGF-1 receptors in various tissues [12]. It is worth noting that IGF-1, produced locally by the bone, was shown to stimulate the activity of 1α-hydroxylase, which regulates formation of active 1,25-dihydroxyvitamin D [4]. Local 1,25(OH)2D3 synthesis and activity is a common feature of human osteoblasts, with a critical role in the development, differentiation, and mineralization of bone tissue [25]. The expression of the vitamin D receptor and the activity of 1α-hydroxylase in bone cells suggests that the level of 1,25(OH)2D may undergo local regulation [23].

The results of our study should be interpreted in the light of some limitations. For technical reasons, we were unable to perform bone mineral density measurements and collect reports on dietary calcium and vitamin D intake. However, the study excluded underweight children (BMI < 5 percentile) and those taking vitamins and medicines that might affect vitamin D levels or bone metabolism. Another limitation of our study was the lack of data on PTH levels. Likewise, we did not measure 1,25(OH)2D concentrations and assessed only the 25(OH)D level as a functional indicator of vitamin D status. Currently, a total of 25(OH)D is thought to be the best biochemical marker of vitamin D sufficiency, even though 1,25(OH)2D is an active vitamin D metabolite. We measured the serum’s total 25(OH)D, with the assay traceable to the isotope dilution-liquid chromatography/tandem mass spectrometry. This assay is characterized by very low cross-reactivity with the C-3 epimers of the 25(OH)D and 24,25(OH)2D metabolites; the former is of particular importance for the assessment of vitamin D status in children.

Our study group included randomly selected healthy, gender-matched, prepubertal school children, aged 9–11 years. It is important that we measured two bone turnover markers, P1NP and CTX-I, recommended as reference markers by the International Federation of Clinical Chemistry and Laboratory Medicine (IFCC) and the International Osteoporosis Foundation to measure when assessing bone turnover in adults but nowadays frequently applied by several researchers investigating bone metabolism in children [19,21,24,26]. In our study group, concentrations of assessed bone turnover markers were similar to those recorded by other investigators in children of the same age range [21,27,28]. Levels of these bone markers are quite stable in this narrow age range, with this being the strength of our study.

A recent study, performed in a large population of children and adolescents to establish reference intervals for bone turnover markers, investigated the influence of obesity and pubertal status on the concentrations of bone markers, PTH and 25(OH)D [26]. In this study, which included the cohort of over 300 obese subjects, it was clearly demonstrated that the concentrations of bone markers and 25(OH)D were significantly lower in obese children, while PTH was significantly increased. Detailed analysis revealed, the levels of P1NP and CTX were the highest in boys at 13 years of age, whereas, in girls, both markers reached the pubertal peak at 10 and 11 years, respectively [26].

Of note, the findings on the levels of bone markers in young obese subjects are contradictory, some of them indicating lower concentrations in the obese [21,29].

Our study group was relatively small but included prepubertal children with normoglycemia aged 9–11 years, of which 11% (14 participants) were obese and less than 5% were in Tanner stage 3. A small number of obese participants, in our opinion, could not influence the reported findings. Furthermore, we did not find associations between the BMI percentile and the markers of bone turnover and 25-hydroxyvitamin D, except the positive weak correlation with IGF-1. In fact, in the presented study, the levels of P1NP were the highest, with the 25(OH)D concentration below <20 ng/mL, and those of CTX were similar, regardless of vitamin D level.

Despite the high prevalence of vitamin D insufficiency and deficiency, screening for 25-hydroxyvitamin D in the pediatric population is currently not recommended, except for individuals who present risk factors for hypovitaminosis D. With a lack of consensus over the optimal cut-off for vitamin D sufficiency in the pediatric population, it is difficult to define that the serum total of 25(OH)D is the best measure of vitamin D status. It is plausible that 25(OH)D level may not necessarily be an adequate predictor of bone turnover in children. Children are not small adults, and it is likely that a real deficiency of vitamin D could be better estimated by other potential markers of vitamin D metabolism. To gain a better understanding of the complexities of vitamin D metabolism in a general population, more research is needed that includes other additional functional metabolites, such as 1,25(OH)2D, 24,25(OH)2D, or, eventually, bioavailable 25(OH)D [30].

The present study demonstrated that low levels of circulating 25(OH)D (<20 ng/mL) do not unfavorably impact bone turnover in children. By contrast, the role of IGF-1 as a strong, positive predictor of bone formation was highlighted. Our study indicated that the measurement of serum 25(OH)D during prepuberty, the period of rapid bone growth and mineralization, offers no additional predictive value for the assessment of bone health in presumably healthy children.

In the light of the latest scientific knowledge, further research is necessary to establish the best marker of vitamin D sufficiency and optimal vitamin D levels that benefit bone health, in order to improve strategies for the prevention of hypovitaminosis D and its consequences for children.

## Figures and Tables

**Table 1:** General characteristics of study subjects.

Characteristics	Girls	Boys	*p*
	*n* = 66	*n* = 62	
Age, years (SD)	9.7 (0.8)	9.8 (0.8)	0.439
Height, cm (SD)	141 (7.8)	145 (9.0)	0.013
BMI, percentile (SD)	54 (30)	53 (33)	0.966
BMI, kg/m^2^ (SD)	18.0 (3.3)	18.7 (4.1)	0.733
25(OH)D, ng/mL (SD)	21.9 (4.9)	21.9 (3.9)	0.917
IGF-1, ng/mL, Me (Q1, Q3)	237 (167, 298)	184 (148, 222)	0.002
CTX, ng/mL, Me (Q1, Q3)	1.50 (1.18, 2.01)	1.51 (1.05, 1.97)	0.623
P1NP, ng/mL, Me (Q1, Q3)	1414 (1224, 1576)	1307 (1215, 1450)	0.156
BTindex, Me (Q1, Q3)	0.31(–0.23,0.95)	0.11(–0.80, 0.56)	0.038

Abbreviations: SD: standard deviation; Me (Q1, Q3): median (quartile 1, quartile3); BMI: body mass index; 25(OH)D: 25-hydroxyvitamin D; IGF-1: insulin-like growth factor 1; CTX: C-terminal telopeptide of type I collagen; P1NP: N-terminal propeptide of type I procollagen; BTindex: bone turnover index; p: statistical significance.

**Table 2:** Participants characteristics by tertiles of serum 25(OH)D.

Characteristics	25(OH)D Tertiles (ng/mL)			
	1.	2.	3.	*p*
	≤19.4	20.1–23.3	23.4–35.3	
	*n* = 39	*n* = 46	*n* = 43	
Girls/boys (N)	23/16	21/25	22/21	
Height, cm (SD)	143 (9)	144 (10)	143 (7)	0.731
BMI, percentile (SD)	54 (30)	53 (31)	52 (34)	0.955
Obesity (N/%)	4/10.0	5/11.0	5/11.0	
IGF-1, ng/mL, Me (Q1, Q3)	225 (176, 260)	201 (153, 250)	189 (151, 243)	0.080
CTX, ng/mL, Me (Q1, Q3)	1.59 (1.21, 2.39)	1.45 (1.03, 2.02)	1.50 (1.05, 1.92)	0.210
P1NP, ng/mL, Me (Q1, Q3)	1415 (1237, 2964)	1392 (1225, 1530)	1299 (1156, 1446)	0.048
BTindex, Me (Q1, Q3)	0.15 (–0.74, 0.95)	0.31 (–0.32, 0.71)	0.21(–0.50, 0.70)	0.763

Abbreviations: SD: standard deviation; Me (Q1, Q3): median (Quartile 1, quartile3); BMI: body mass index; 25(OH)D: 25-hydroxyvitamin D; IGF-1: insulin-like growth factor 1; CTX: C-terminal telopeptide of type I collagen; P1NP: N-terminal propeptide of type I procollagen; BTindex: bone turnover index; p: statistical significance.

**Table 3:** Correlation analysis among study subjects.

	IGF–1	25(OH)D
IGF–1, R (*P*)		–0.180
		(0.042)
P1NP, R (*P*)	0.505	–0.168
	(<0.001)	(0.059)
CTX, R (*P*)	0.221	–0.085
	(0.012)	(0.338)
BTI, R (*P*)	0.231	–0.055
	(0.009)	(0.535)
BMI, percentile R (*P*)	0.216	–0.129
	(0.014)	(0.147)

Abbreviations: IGF-1: insulin-like growth factor 1; 25(OH)D: 25-hydroxyvitamin D; P1NP: N-terminal propeptide of type I procollagen; CTX: C-terminal telopeptide of type I collagen; BTindex: bone turnover index; r: correlation coefficient; p: statistical significance.

**Table 4:** Linear regression analysis for the prediction of bone turnover markers and IGF-1 variation by low 25(OH)D (<20 ng/mL) in study subjects.

Model	Independent Variable	R2	B (Standardized)	*p*
Model 1.				
P1NP		0.059		0.050
	25(OH)D		0.199	0.025
Model 2.				
CTX		0.052		0.080
	25(OH)D		0.155	0.080
Model 3.				
IGF-1		0.149		<0.001
	25(OH)D		0.185	0.036

## Supplementary Materials

The following are available online at https://www.mdpi.com/article/10.3390/nu13103324/s1, Table S1: Characteristics of study participants after partition in tertiles according to IGF-1 concentrations.
