# Supplementary material for: Low Serum 25-hydroxyvitamin D Level Does Not Adversely Affect Bone Turnover in Prepubertal Children
Source: Nutrients. 2021 Sep 23;13(10):3324. doi: 10.3390/nu13103324 (PMC8537639; doi:10.3390/nu13103324)
Supplement: Supplementary file 1 [file nutrients-13-03324-s001.zip › nutrients-1370512-SI.pdf]

Supplementary Material

Table S1. Characteristics of study participants after partition in tertiles according to IGF-1 concentrations.

**Characteristics**	**IGF-1 tertiles (ng/mL)**			
	**1.**<br>≤ 169.3<br>N=42	**2.**<br>172–229<br>N=43	**3.**<br>230–541<br>N=43	***P***
Height, cm (SD)	141 (8)	144 (8)	145 (9)	0.043
BMI, percentile (SD)	44 (31)	56 (32)	59 (30)	0.056
25(OH)D ng/mL (SD)	23.1 (4.0)	21.7 (4.3)	21.3 (4.4)	0.106
CTX, ng/mL, Me (Q1, Q3)	1.50 (1.06, 1.83)	1.50 (1.17, 2.26)	1.50 (1.05, 1.92)	0.126
P1NP, ng/mL, Me (Q1, Q3)	1225 (1116, 1358)	1357 (1240, 1511)	1497 (1303, 3012)	<0.001
BT index, Me (Q1, Q3)	0.08 (–0.53, 0.59)	0.15 (–0.76, 0.56)	0.52(–0.10, 1.71)	0.045

Abbreviations: N: number; IGF-1: insulin-like growth factor 1; SD : standard deviation; Me (Q1, Q3) : median (Quartile 1, quartile3); BMI: body mass index; 25(OH)D :25-hydroxyvitamin D; CTX : C-terminal telopeptide of type I collagen; P1NP: N-terminal propeptide of type I procollagen; BTindex: bone turnover index; p:statistical significance
